# Systematic conservation planning for nature recovery

**DOI:** 10.1093/biosci/biaf030

**Published:** 2025-04-23

**Authors:** David J Baker, Kevin J Gaston, Kristian Metcalfe, Ilya M D Maclean

**Affiliations:** Environment and Sustainability Institute; Environment and Sustainability Institute; Centre for Ecology and Conservation, University of Exeter, Penryn, Cornwall, England, United Kingdom; Environment and Sustainability Institute

**Keywords:** ecosystem restoration, spatial prioritization, biodiversity

## Abstract

Nature conservation is increasingly focused on recovering depleted populations and ecosystems. The United Nations General Assembly has proclaimed 2021–2030 the UN Decade on Ecosystem Restoration, and global commitments to ecosystem restoration in response to biodiversity, climate, and sustainable development targets are now considerable, with over 100 nations committed to halting and reversing forest loss and land degradation by 2030. The impacts of these resources on nature recovery will depend on how actions are identified and implemented. Systematic conservation planning has historically been used to prioritize areas for protection but has shown great potential to guide nature recovery actions that are underpinned by principles of spatial conservation planning. In the present article, we advocate for systematic conservation planning to target resources for nature recovery and show how well-established systematic conservation planning frameworks can be developed appropriately, particularly by integrating models for forecasting ecological, social, and economic conditions with spatial prioritization methods designed to target nature recovery resources.

The United Nations General Assembly has proclaimed 2021–2030 as the UN Decade on Ecosystem Restoration (Resolution 73/284), with the objective to prevent, halt, and reverse the degradation of ecosystems worldwide: The decade is underway, and the stakes are high for global biodiversity (WWF 2022). The targets agreed on at the 15th United Nations Biodiversity Conference recognize the importance of restoration in “substantially increasing the area of natural ecosystems by 2050” (CBD 2022). The Glasgow Leaders’ Declaration on Forests and Land Use, established during the 26th United Nations Climate Change Conference, commits over 100 nations to collaborate in halting and reversing forest loss and land degradation by 2030. Furthermore, additional efforts to restore ecosystems have been set forth through national commitments within the Paris Climate Agreement, aimed at limiting global warming to 2 degrees Celsius, and the 2030 Agenda for Sustainable Development, which seeks to halt and reverse land degradation. These commitments build on work undertaken in response to the Bonn Challenge, which has received pledges from over 60 countries to deliver collectively more than 200 million hectares of restoration on degraded and deforested land and aims to reach 350 million hectares by 2030 (Dave et al. 2018).

Biodiversity losses in most biomes have been substantial (WWF 2022), which has driven breakdowns in ecological processes and associated ecosystem services (IPBES 2019). Land- and sea-use change, the major direct driver of recent biodiversity loss (Jaureguiberry et al. 2022), is ongoing, with a 9% increase in global croplands between 2000–2019—half this area replacing natural vegetation (Potapov et al. 2022)—and approximately 5 million hectares per year of forest loss over the same period (FAO 2021). Although this emphasizes the need to protect the remaining areas of natural vegetation, reversing declines in biodiversity and restoring ecological processes requires not only the prevention of further losses but, where possible, the restoration of degraded ecosystems and the creation of new habitat for species on converted lands (Anderson 1995, Possingham et al. 2015, Leclère et al.^ 2020^, Garibaldi et al. 2021). This objective can be broadly termed *nature recovery*. Although there is a degree of ambiguity associated with the meaning of nature recovery and similar terms, we define it in line with the International Principles and Standards for the Practice of Ecological Restoration, which reserves “the term *restoration* for the activity undertaken and recovery for the outcome sought or achieved” (Gann et al. 2019). Nature recovery is not defined or measured simply in terms of coverage targets for revegetation (e.g., afforestation) but rather in terms of the quantifiable outcomes for specific species (e.g., population size or area occupied), vegetation types (e.g., coverage and quality), or processes (e.g., through restoring ecological functions, such as seed dispersal by frugivores). We use *nature recovery* rather than *biodiversity recovery* to reflect that targeted outcomes may be broader than biodiversity, although species conservation is often a major focus of nature recovery efforts. The focus of and specific objectives for nature recovery, such as when a population or ecological process is deemed to have recovered, are context specific and will often be influenced by regional, national, and global contexts, but targets and action plans for nature recovery are now widely incorporated into policy (e.g., EU Biodiversity Strategy for 2030; EC 2020).

Although the opportunities for nature recovery are promising, too often, resources designated for ecosystem restoration result in on-the-ground actions that do not contribute adequately toward nature recovery, such as planting monocultures of nonnative trees (Seddon et al. 2019), afforestation of land best suited to other vegetation types (Bond et al. 2019), or restoration of vegetation vulnerable to climate change (e.g., Harris et al. 2006). This raises concerns that the resources pledged to ecosystem restoration will be less effective at delivering nature recovery outcomes than they might have been (Hua et al. 2022) and will miss potential synergies between actions taken toward nature recovery objectives and those primarily targeting particular ecosystem services (e.g., nature-based solutions), such as the increasing demand for afforestation to sequester carbon (Carwardine et al. 2015, Strassburg et al. 2019, Aguirre-Gutiérrez et al. 2023). Although opportunities are being missed, decades of systematic conservation planning experience could be drawn on to target available resources more effectively toward delivering nature recovery outcomes, inclusive of protecting, restoring, and creating new high-value nature areas, especially in working landscapes with multiple, competing demands on land (Garibaldi et al. 2021).

Systematic conservation planning emerged as a structured, participatory approach for guiding objective and evidence-based decisions about the types and locations of conservation action (Margules and Pressey 2000, Knight et al. 2006, McIntosh et al. 2017). It is most strongly associated with protected area network design, where the objective is to establish or expand networks across areas of natural vegetation to support the long-term persistence of conservation features (e.g., species or vegetation communities) in landscapes (see table 1 for glossary of key systematic conservation planning terms; Fernandes et al. 2005). Systematic conservation planning is target driven, informed by spatial information on conservation features, with targets representing aspects of population viability (e.g., the minimum area of occupancy) or the amount of each vegetation or habitat type required to ensure a desirable fraction of each species’ occupied range or population is protected. Systematic conservation planning then relies on selection processes or algorithms that typically exploit differences in species composition between sites (i.e., complementarity) and account for between site measures of connectivity and economic costs of protecting or managing land (Kukkala and Moilanen 2013) to find subsets of locations that collectively satisfy conservation goals (Margules and Pressey 2000, Kukkala and Moilanen 2013, McIntosh et al. 2017). Despite some debate about the realized impacts (McIntosh et al.^ 2017^, ^2018^), systematic conservation planning has had a strong influence on terrestrial and marine protected area network design globally (Fernandes et al. 2005, Álvarez-Romero et al. 2018). Nevertheless, the influence of systematic conservation planning on the targeting of nature recovery opportunities appears limited in practice across much of the globe, despite evidence for large increases in the cost effectiveness when resources are allocated strategically (Strassburg et al.^ 2019^, ^2020^).

**Table 1.** Glossary of key systematic conservation planning terms.

Term	Definition
Planning region	Spatially defined area within which conservation actions are designed and implemented. A region might be defined, for example, by management or political boundaries or by biogeographic characteristics.
Planning unit	The spatial unit serving as the basic building block for designing and implementing conservation strategies and prioritizing conservation actions within a planning region.
Conservation features	A specific element of biodiversity or an ecological attribute for which actions are targeted in conservation planning processes. Features may be species, but also vegetation or assemblage types or even geographic features and processes.
Surrogates	An ecological feature used as a replacement for a more intricate or challenging to measure target, enabling conservation planners to make informed decisions when direct assessment or monitoring of specific biodiversity elements is impractical or challenging.
Goals	A vision statement that steers the development of conservation plans and strategies, typically focused the desired effect on biodiversity, or ecosystem function or services, inclusive of human health and wellbeing.
Targets	Specific, quantifiable objectives for conservation features, likely set to ensure persistence of the feature in the planning region (e.g., ensure minimum viable population sizes). Typically, proxies for actual population estimates are used, such as area weighted number of planning units in which the feature occurs, because they are easier to quantify.
Representation	The extent to which a comprehensive set of conservation features is adequately included within a conservation plan or network. It ensures that a range of conservation features are sufficiently covered to maintain the overall biodiversity and ecological integrity of the planning region.
Complementarity	The principle of selecting conservation actions or areas that contribute unrepresented features to a portfolio of sites. The objective is to maximize the effectiveness and efficiency of conservation efforts within a designated planning region.
Efficiency	The optimization of resource allocation to achieve conservation goals with minimal resources and effort, maximizing benefits while minimizing negative impacts or trade-offs, thereby ensuring the effective use of limited resources and time.
Conservation assessment	The process of prioritizing new conservation actions. This is typically achieved using mathematical decision-theory methods that seek efficient solutions to prioritization problems.
Minimum set problem	The computational challenge of identifying the smallest set of areas needed to achieve the specified conservation goals, aiming to meet all biodiversity targets, where possible, while minimizing costs and trade-offs.
Maximal coverage problem	The computational challenge of selecting areas that collectively provide the highest representation of biodiversity features within budget and spatial constraints of the planning activity.
Costs	Costs encompass various expenses, trade-offs, or negative impacts associated with conservation actions, including financial expenditures, opportunity costs, and environmental and social impacts.
Restoration gaps	The disparity or deficiency in the representation and protection of degraded biodiversity features, including temporal gaps in feature representation caused by time lags in habitat maturation after conservation action.

Although the basic structure of systematic conservation planning is not changed when used in a nature recovery context, many aspects of the problem framing are fundamentally altered, which has important consequences for each systematic conservation planning stage (box 1) and affects the information requirements for conducting spatial prioritizations (figure 1). This can be understood by considering that nature recovery is by necessity focused on land that is considered to be no longer contributing toward valued outcomes for nature (e.g., intensively managed agricultural land). In these areas, the historical abiotic conditions, vegetation types, species assemblages, and ecological processes once present are often uncertain or unknown or may no longer be relevant. By introducing a range of choices for what action to take, the complexity of the spatial prioritization process increases greatly, which would conventionally just be limited to a binary choice (protect or not). Furthermore, the difference between the initial state and the target state of the land will typically be greater for actions focused on habitat creation, resulting in substantial time lags between interventions and targeted outcomes, along with less certainty about achieving those outcomes. Emphasis therefore must be placed on anticipating the effects of significant changes in the amount, type, and spatial arrangement of resources and conditions in the landscape on nature recovery outcomes, and prioritizing decisions against outcomes that may take decades to be realized.

**Figure 1.**
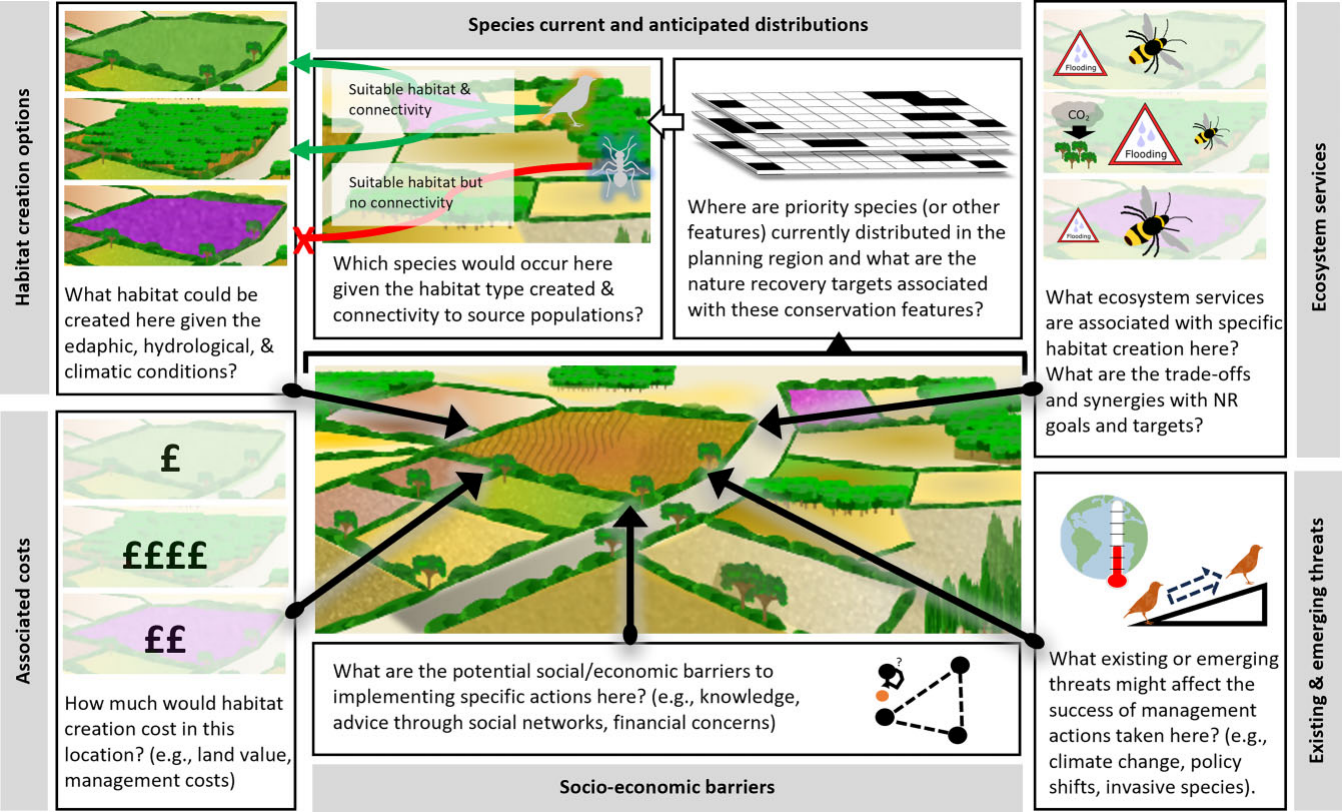
Systematic conservation planning in a nature recovery context requires substantial information on current and future conditions in each planning unit, particularly in multifunctional and highly modified landscapes. Information is required on potential nature recovery actions permissible in each planning unit (in the present figure, a field parcel) on the basis of edaphic, hydrological, and climate conditions. Conservation features must be mapped and potential responses to specific actions anticipated, accounting for population processes that affect colonization likelihood from source populations. Information on threats, costs, and potential synergies and trade-offs with ecosystem services—such as flood risk reduction, carbon sequestration, pollination—are among the additional information required to prioritize nature recovery actions in complex socioecological landscapes.

In this article, we propose that achieving the objectives of the UN Decade on Ecosystem Restoration requires building on lessons learned through systematic conservation planning to guide nature recovery. Systematic conservation planning has undergone extensive development over many decades, with well-established frameworks offering opportunities to guide nature recovery projects from conception to implementation. In the present article, we discuss how these frameworks can be developed in a nature recovery context to incorporate principles and data unique to the challenges of delivering nature recovery in complex and dynamic anthropogenic landscapes. We draw attention to the anticipatory nature of systematic conservation planning for nature recovery, where conservation decisions must be based on predicted responses of biodiversity features to management actions, including creation of habitat for species on converted land. We discuss approaches for solving problems in spatial prioritization and optimization in a nature recovery context and discuss the potential for adapting and developing spatial prioritization methods tailored to specific needs of nature recovery found in varying landscape contexts across the globe. Finally, we highlight several key challenges to adopting systematic conservation planning as best practice for targeting resources toward delivering ambitious global ecosystem restoration and nature recovery targets.

## The process of systematic conservation planning

Systematic conservation planning is traditionally divided into stages, each with a distinct purpose. Although the exact structure of systematic conservation planning varies depending on the planning scenario, the 11-stage systematic conservation planning framework from Pressey and Bottrill (2009) encompasses the core stages traditionally included in the process. This framework expands earlier versions (e.g., Margules and Pressey 2000) to increase the emphasis placed on the social and economic elements that are integral to successful conservation planning in anthropogenic landscapes, elements that are also critical to the effective delivery of actions targeting nature recovery. The major focus of traditional systematic conservation planning has been on prioritizing areas of existing habitats or vegetation types in the process of creating or expanding protected area networks, where the process is targeting the protection of conservation features (species or vegetation types) in areas of the landscape that they currently occupy. Throughout, we label this context *nature protection*.

Systematic conservation planning starts by defining the conservation planning problem, including the geographic scope of the planning region and the overall goals of the conservation planning activity. The planning region is often defined by geopolitical boundaries, but other strategies include defining the region on the basis of environmental (e.g., hydrological catchments) or biogeographic characteristics. The overall goals of the conservation planning activity are defined as broad, qualitative statements about the biodiversity and societal objectives (Pressey and Bottrill 2009). These goals should be aligned with policy and stakeholder input, representing a range of community voices and shared values and may encompass diverse ways of valuing nature and its impact on society (Knight et al. 2006). At this initial stage, available financial resources and the required practitioner skills are identified (Watson et al. 2011). Stakeholder engagement from the start is important to understand the socioeconomic conditions of a planning region, which strongly affect conservation actions and priorities (Naidoo et al. 2006, Pressey and Bottrill 2009). Conditions encompass local livelihoods, economic reliance on natural resources, cultural values, and legal factors affecting planning decisions (Ban et al. 2013). When used in a nature protection context, key conditions are those that affect the protection of land that has existing or potential high value for nature, including the cost of acquiring and managing land. Integrating socioeconomic data into systematic conservation planning helps balance conservation objectives with stakeholders’ concerns, leading to more feasible, sustainable, and equitable outcomes (Knight et al. 2006, Ban et al. 2013, McIntosh et al. 2017).

The next major stage of systematic conservation planning involves gathering information on the current spatial distribution of conservation features, which is crucial for quantifying the contribution of planning units to conservation goals. Where species are the conservation features, distribution mapping is usually highly incomplete, particularly at high spatial resolutions, and relying solely on known occurrences is undesirable because of major data gaps and uncertainties associated with the presence and absence of species across planning units (Baker et al. 2021). To overcome data gaps, outputs from species distribution models are commonly used in systematic conservation planning (Thomson et al. 2009), but alternatively, broadscale surrogates of species distribution, such as vegetation types or landscape structural complexity, can be used to capture the distribution of biodiversity on the basis that representing a given proportion of vegetation or structure types will protect a percentage of species (e.g., defined by species–area relationships; Desmet and Cowling 2004). Ultimately, data must be identified to serve as surrogates for the broader suite of conservation features in the landscape (Rodrigues and Brooks 2007, Hanson et al. 2017).

Box 1. **Stages in systematic conservation planning for nature recovery.**

Adapting the 11 stages from Pressey and Bottrill (2009) to a nature recovery context where text in italics indicates key differences, building from Margules and Pressey (2000).

**Stage 1: Define the scope and cost the nature recovery activity**

Define boundaries of planning region. Determine the budget, *including sources of long-term funding to support nature recovery activities*. Identify skills required by the systematic conservation planning team.

**Stage 2: Identify and involve stakeholders**

Identify key stakeholders and engage disengaged individuals or communities. Identify contentious activities, particularly where *nature recovery activities may require major changes in land use or characteristics, including species composition (e.g., species eradications or reintroductions)*.

**Stage 3: Describe the socioeconomic–cultural context for nature recovery**

Describe the social, economic, and political setting. *Consider stakeholder attitudes to nature recovery, legal frameworks, and financial mechanisms (e.g., environmental subsidies and credit markets), with emphasis on securing long-term investments*.

**Stage 4: Identify nature recovery goals**

Create a vision statement, capturing broad representative values, *including statutory nature recovery goals and regional and local priorities*. These may capture ecological (e.g., stable populations) and ecosystem services (e.g., flooding, human well-being). *Emphasis is placed on ambitious visions for building back resilient ecosystems*.

**Stage 5: Compile data on socioeconomic variables, threats, and potential areas for nature recovery**

Data on costs of activities (e.g., opportunity/management costs). Data on social or political *opportunities and barriers to engagement with nature recovery*. Continuing and potential threats to biodiversity, including *forecasts of emerging threats. Identify areas available for nature recovery, quantifying site condition, potential target states, and the likelihood of achieving these states under relevant socioeconomic scenarios*.

**Stage 6: Compile data on the conservation features of the planning region**

Review existing data, identify features where data quality is sufficient to inform systematic conservation planning and *where responses to nature recovery action can be forecast with some confidence*. Identify surrogates for features without sufficient data. *Identify nature recovery actions shown to facilitate recovery of target features. Develop quantitative information to anticipate responses of features to nature recovery activity (e.g., habitat suitability models)*.

**Stage 7: Set quantitative targets for nature recovery within the planning region**

Set quantitative targets for conservation features to be achieved at a *future time point as the outcome of nature recovery actions, likely on the basis of functional or historical reference points, often linked to policy targets*. Set quantitative targets for minimum habitat patch size, quantity, quality, or connectivity to be *achieved in the future through targeting of nature recovery opportunities*.

**Stage 8: Review existing conservation areas**

Estimate the extent to which quantitative targets have been achieved by existing protected areas and areas of natural habitat. Identify emerging threats to existing areas, *including climate change, invasive species, and socioeconomic trends*.

**Stage 9: Select new nature recovery areas and actions (conservation assessment)**

Select new areas and specific nature recovery actions in those areas (e.g., woodland creation, wetland restoration) *that contribute toward nature recovery targets, with this contribution identified through forecasted responses of features to nature recovery actions*. Identify the temporal scheduling of activities and trajectories toward targets considering restoration gaps. Agree nature recovery actions with stakeholders, evaluating trade-offs between different choices.

**Stage 10: Implement nature recovery management actions in selected areas**

Engage with land managers to determine appropriate form of management. Build stakeholder networks (e.g., landowners, community) to facilitate joined up nature recovery activity. *Build community support for nature recovery recognizing that large changes might affect (positively and negatively) some existing activities (e.g., farming, recreation)*. Ensure legal protections and management solutions are in place to buffer nature recovery from external conditions (e.g., market forces, social trends) over sufficient time scales for nature recovery outcomes to be realized.

**Stage 11: Maintain the required value of conservation and nature recovery areas**

Set nature recovery monitoring targets that recognize *realistic timescales for reaching targets, incorporate intermediate objectives to assess progress at earlier time points*. Design monitoring protocols for measuring the progress toward targets. Conduct appropriate management to ensure targets are met. Periodically evaluate overall performance of nature recovery activities, identifying weaknesses and opportunities for improvement.

Setting quantitative targets for conservation features involves converting broad goals into objective criteria that can be evaluated quantitatively. In a nature protection context, targets typically aim for continued feature persistence (e.g., stable populations) in the planning region while ensuring representation of all features across a portfolio of protected areas. The targets are often defined by the minimum number of sites or area each feature should constitute (e.g., vegetation types) or occupy (e.g., species) across the planning region. Ideally, the targets would be set on the basis of detailed ecological analysis—for example, to establish minimum viable population sizes and estimates of habitat-specific population densities. Rarely are these data available, and targets are often set without a strong ecological rationale, typically on the basis of heuristic rules (e.g., expert opinion) or policy objectives (e.g., protecting a percentage of the area of a species’ habitat). Approaches linking habitat area targets to species traits, such as using allometric scaling relationships between body size and population density to estimate the habitat area requirements for a species, have been used as more ecologically grounded alternatives to heuristic methods (Pressey et al. 2003). The targets might also consider characteristics of patch size and quality that affect occupancy, including setting a minimum patch size (Smith et al. 2010), demographic connectivity such that selected areas form viable metapopulations (Daigle et al. 2020), or being weighted on the basis of species- or trait-specific threats or the rarity of conservation features (Chan et al. 2006, Gordon et al. 2009). Some conservation features may not meet the identified targets (i.e., they are not present in a sufficiently high number of protected sites), requiring the addition of new sites to the protected area portfolio to meet shortfalls. Target setting has significant consequences for planning, which places great importance on understanding the consequence of using any particular strategy (Pressey et al. 2003, Rondinini and Chiozza 2010).

Conservation assessment, the process of prioritizing new conservation actions, comprises its own distinct steps, including dividing a planning region into planning units, quantifying the amount of each conservation feature and the socioeconomic costs of protection per planning unit, assigning targets and weights to features that reflect priorities, and then running a spatial prioritization analysis to meet specified targets. Setting the planning unit size can have important effects on spatial prioritizations and therefore requires careful consideration. Conservation features are mapped to planning units, and therefore, changing the size of the unit can alter the apparent spatial distribution of the feature (Nhancale and Smith 2011). The planning unit size should reflect the confidence in the accuracy of conservation feature data while also recognizing that smaller planning units are likely to better capture areas of habitat in fragmented landscapes and barriers to species movements between patches, and they generally result in more efficient solutions because each unit contains fewer nontarget areas (Nhancale and Smith 2011). Several computational or decision support tools are available to solve prioritization problems, although the precise framing of the problem differs among those tools. The most common and widely used heuristic approaches for identifying priorities are Marxan and Zonation. Marxan aims to meet specified targets while minimizing costs (minimum set problem; Ball et al. 2009), whereas Zonation aims to maximize biodiversity gains for a given cost (the maximal coverage problem). Although these two widely used tools can only find near optimal solutions, mixed integer linear programming (e.g., prioritizr; Hanson et al. 2024) can find optimal solutions and incorporate different management actions or zones. Each of these tools has been used in systematic conservation planning, with each having different strengths and weaknesses, and most can accommodate core conservation principles by emphasizing to some degree the importance of, for example, patch size and connectivity, and the incorporation of information on threats, costs, and opportunities. The principal choice of prioritization algorithms centers on the framing of the prioritization problem, whether the algorithm is guaranteed to find efficient solutions, and the computational resources required to solve the prioritization problem (Delavenne et al. 2012, Beyer et al. 2016).

The outputs from conservation assessment are designed to guide decisions about which new areas to add to an existing protected area portfolio rather than to be definitive. A key step involves stakeholder evaluation, which can take a wealth of approaches, but key is highlighting trade-offs of adopting different strategies to identify outcomes that better balance conservation objectives and stakeholder interests and reconciling disagreement between prioritization outputs and on-the-ground perspectives (Klein et al. 2010, Metcalfe et al. 2015). The scheduling of implementation is agreed, informed by data on site irreplaceability and risks of delaying action (e.g., potential for logging). Finally, to accrue benefits from systematic conservation planning requires effective management that relies on robust monitoring strategies and the development of socioecological performance indicators to evaluate changes over time relative to established benchmarks.

## Adapting systematic conservation planning to a nature recovery context

In this section, we highlight key conceptual and methodological elements relevant to each systematic conservation planning stage when applied to nature recovery. These are discussed with reference to box 1, which reframes the 11 stage systematic conservation planning framework presented by Pressey and Bottrill (2009) in a nature recovery context. Many elements are relevant for both nature protection and recovery context, but the contexts can have important effects on the types of information required and how this is used within systematic conservation planning, and we draw out these distinctions throughout. We also highlight the diverse range of information and potentially complex spatial prioritization process of nature recovery systematic conservation planning (figure 1) and reflect on existing examples of systematic conservation planning applied to problems with nature recovery objectives (table 2). These examples are used to highlight the limitations with existing approaches and to identify key advances needed to overcome these challenges.

**Table 2.** Examples of systematic conservation planning approaches used for targeting nature recovery.

Study	Objective	Targets	Nature recovery actions	Anticipated responses	Costs	Threats	Prioritize actions
Westphal and colleagues (2007)	Optimal landscape restoration for suite of bird species.	Maximize the summed probability of occurrence over all species and revegetation sites given a budget size	Revegetate to historical coverage	Species distribution models based on historical species records and vegetation coverage	Linear function of property value	None	Simulated annealing with custom objective function.
Thomson and colleagues (2009)	Spatial/temporal revegetation priorities to maximize habitat for birds (balanced solutions, no species doing poorly).	Rank sites by expected contribution to future biodiversity gain (habitat suitability)	Revegetation (recreate original state)	Occupancy models (habitat predictors)	None	None	Zonation
Strassburg and colleagues (2019)	Maximize ecosystem service benefits of forest restoration (biodiversity or carbon sequestration).	Scenarios with varied weightings applied to biodiversity and carbon targets, and cost constraints.	Proportion of forest historic extent to restore	Forecast extinction risk based on the species–area relationship, with potential distributions assuming restoration inferred using species distribution models; benefit of restoration assuming diminishing returns of adding more habitat to a unit.	Restoration uncertainty costs × planting costs + fencing costs.	None	Linear programming
Gilby and colleagues (2021	Prioritize restoration to improve habitat quality.	Restore habitat matrix to increase total fish/harvestable fish abundance	Seagrass, oyster reef, mangrove restoration	Modelled relationship between fish abundance and extent of each habitat type	None	None	Bayesian belief network.
Shoo and colleagues (2021)	Schedule restoration to achieve maximum quality gain within budgetary constraints.	Scenario based, linked to perceived biodiversity value of being in each habitat state.	Restoring habitat across four discrete habitat states.	Scenario-based, assuming different biodiversity benefits of transitioning from one state to another and different timescales for habitat succession	Diminishing restoration costs through time, accounting for variations with site characteristics (e.g., accessibility).	None	Integer linear programming
Mu and colleagues (2022)	Restoration trade-offs of ecosystem services to maximize cobenefits.	Multiple scenarios for restoration area.	Restore farmland to forest or wetland (based on soil features or topography).	Four ecosystem services (carbon storage, soil retention, water yield, habitat quality); benefits calculated using a natural capital model. Habitat quality incorporates measures of threat and vulnerability to threats.	Opportunity (from cultivated land) and restoration (e.g., engineering) costs.	Via natural capital model	Marxan
Smith and colleagues (2022)	Identify a potential nature recovery network including core and recovery zones, with the latter managed to improve ecological conditions.	Expert set habitat-type targets.	Indirectly	Indirectly by including targets for habitat-types that could be restored.	Agricultural land quality	None	Marxan
Cattarino and colleagues (2015)	To prioritize the set of actions to address threats to freshwater fish species that achieves the conservation target at minimal cost.	Specific combination of actions necessary to remediate threats to species.	Multiple actions with potential to remediate threats to target species.	Species-specific responses to threats abatement, e.g., obtained from literature or plausible assumptions.	Land acquisition costs.	Considered as actions in this analytical framework.	Bespoke multiaction prioritization algorithm

### Setting nature recovery goals and targets in systematic conservation planning

The first major challenge of implementing systematic conservation planning in a nature recovery context is setting the goals and targets (box 1, stages 4 and 7). Broad nature recovery goals reflect the ambitions and vision of society and must capture both human and nonhuman requirements of landscapes, which will vary in their degree of alignment and will often necessitate resolving strong trade-offs. Achieving many goals will require substantial landscape alteration (e.g., on agricultural land), potentially affecting established human–landscape relationships, especially in areas influenced strongly by social or cultural relationships to land (e.g., Iversen et al. 2022). Because nature recovery can be disruptive and contentious (Wynne-Jones et al. 2018); for example, where changes in farming practices or species reintroductions or removals are involved, early identification and mediation of issues through stakeholder engagement are crucial to developing nature recovery goals (box 1, stage 2). The goals must consider potentially diverse stakeholder perspectives, such as varying perceptions of the need for and effectiveness of nature recovery actions (Mikołajczak et al. 2022, Dunn-Capper et al. 2023), and must identify opportunities to cooperate effectively to improve overall nature recovery outcomes (e.g., O'Bryan et al. 2023). These disparities in values and priorities are not only natural but also central elements of the planning process. Systematic conservation planning can have an important role in finding solutions where differences in stakeholder priorities may require trade-offs (e.g., nature recovery versus carbon sequestration; Strassburg et al.^ 2019^, ^2020^), but it is imperative to address these differences from the outset and work toward synthesizing them into a unified vision for nature recovery that spans the entire planning region. Crafting a shared vision for nature recovery that accommodates and reconciles these divergent perspectives is critical for delivering successful and sustainable nature recovery.

**Figure 2.**
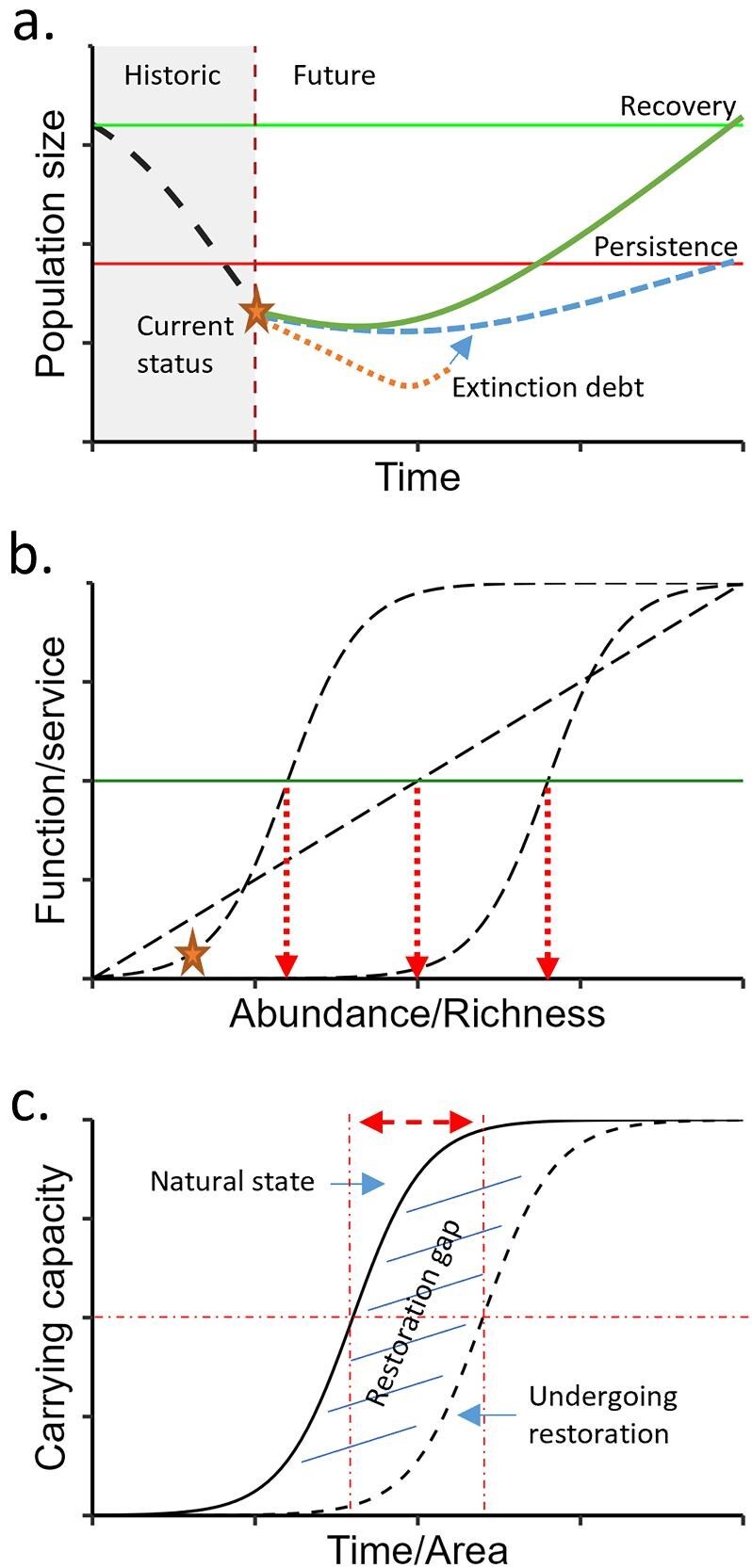
Identifying nature recovery targets on the basis of (a) populations and (b) function and services, and (c) recognizing restoration gaps. Population targets (a) can be benchmarked against historical population sizes (the solid line) or aim to ensure persistence of the species (the lower dashed line). These targets are likely to differ and reaching those targets may need to account for extinction debts that result in continued decline of populations after nature recovery activity is implemented (the dotted line). Ecosystem function or service targets (b) linked to, for example, abundance or species richness depend on these functional relationships (e.g., linear, threshold, logistic). Targets will differ for the same function or service outputs (e.g., restore 50% of historical levels, as pictured) with different functional relationships and understanding these relationships will be important in setting targets. Restoration gaps (c) describe the delay in restored habitat reaching the same quality as habitat in a natural state and, therefore, targets require either more time or a larger area (the dashed double-headed arrow) to support the same amount of conservation features as the natural state habitat.

A critical stage of systematic conservation planning is converting broad nature recovery goals into quantitative targets for conservation features that will be a target of optimization in the conservation assessment stage (box 1, stage 7). The targets for nature recovery are often less clearly defined than those for nature protection (Grace et al. 2019), but recent efforts have begun to be focused on quantitative definitions, such as “a fully recovered species being viable and ecologically functional across their indigenous range” (Akçakaya et al. 2018). Initiatives such as the International Union for Conservation of Nature's (IUCN) Green List propose fixed recovery benchmarks on the basis of historical population status, and several dates have been suggested to define a species as “fully” recovered (e.g., from 1750 to the start of the Industrial Revolution; Grace et al. 2019). Benchmarking nature recovery against historical populations sets aspirational targets but requires caution because of uncertainties in historical population sizes and their attainability given modern constraints on land availability and future climate conditions (Grace et al. 2019). Nature recovery targets should at a minimum support the persistence of species but, where possible, should be more ambitious, acknowledging the scale of historical losses, the perilous state of many species, including the likelihood that extinction debt is highly prevalent in many populations (figure 2a), and the impacts on ecological processes of failing to recover populations (figure 2b). Focusing solely on persistence and threatened species may overlook opportunities to restore ecological functions linked to declines in nonthreatened species (Winfree et al. 2015, Baker et al. 2019). Recovery targets should ideally be informed by knowledge of abundance–function relationships, which may indicate that targets for conservation features need to be set higher to restore ecological function than to ensure persistence (figure 2b; Baker et al. 2019).

Species often have the least predictable response to nature recovery because of nonequilibrium community dynamics and hysteresis effects (Cava et al. 2018). Predictability hierarchies generally follow from vegetation structure to taxonomic diversity then functional diversity and, finally, taxonomic composition (Laughlin et al. 2017). Shifting focus up this hierarchy toward greater predictability might be beneficial when there is high uncertainty in species outcomes and when restoring ecosystem processes or services is required. Areas that are of particular importance for “the continued provision of ecosystem functions and services” are emphasized alongside biodiversity in the Kunming–Montreal Global Biodiversity Framework (CBD 2022), and considerable progress has been made in integrating these aspects of biodiversity into systematic conservation planning (Strassburg et al. 2019, Villarreal-Rosas et al. 2020, Mu et al. 2022). Trait-based approaches informed by community assembly theory are increasingly being used to target species groups to recover particular ecosystem processes or services (Carlucci et al. 2020). The targets can be selected on the basis of traits and considering functionally or genetically complementary or redundant species (Noss et al. 2009, Laughlin et al. 2018, Nielsen et al. 2023) and informed by the functional relationships between specific aspects of biodiversity and function or service outputs (figure 2b).

It is important to be clear about what is being targeted in nature recovery systematic conservation planning. Where species are the conservation feature for which targets are set, prioritization problems are typically aiming to protect, restore, or create habitat sufficient to meet targets. *Habitat* refers to the natural environment where species live and is a species-specific concept that encompasses biotic and abiotic conditions. Creating a particular type of vegetation structure or community does not necessarily equate to creating habitat for a particular species, where critical resources or conditions may be absent (e.g., thermal conditions), nor does it equate to particular ecosystem processes or services. Therefore, there is a clear distinction between prioritizing for species- or process-specific targets and, for example, broad vegetation types (e.g., deciduous woodland), and it is important to be specific about the conservation features being assessed.

The targets should also recognize restoration gaps (i.e., a disparity or deficiency in the representation and protection of degraded biodiversity features), where some environments need extended time for recovery, and, therefore, larger spatial extents need to be restored to have the same function as “pristine” areas (figure 2c). The size of the restoration gap will in part reflect the duration of the time lag in achieving the required habitat state after conservation action, as well as the time taken for target conservation features to respond (e.g., based on life-history traits), which might be affected by the spatial configuration of habitat patches through effects on colonization rates (Kapás et al. 2023) or the likelihood of achieving a successful outcome (McBride et al. 2010). Measuring restoration gaps might require empirical data on the differences in species’ abundances across areas of differing restoration states, and this may only be available for a subset of species. Consultation with species experts can be valuable in obtaining qualitative data on potential restoration gaps that could then be used to scale targets for conservation features accordingly.

### Accounting for the socioeconomic context to nature recovery in systematic conservation planning

Changing land use to deliver nature recovery is inextricably linked to socioeconomic factors affecting land value, inclusive of economic (e.g., food production), recreational, and cultural value (Brown et al. 2022). This is the second major challenge of implementing systematic conservation planning in a nature recovery context. Predicted patterns of land-use change, and therefore opportunities for nature recovery, differ substantially depending on assumptions about socioeconomic and climatic conditions and lead to very different outcomes for biodiversity in the absence of directed nature recovery activity (Brown et al. 2022). A major challenge is securing long-term restoration commitments, particularly where external factors can rapidly shift the demands on land (e.g., the Russia–Ukraine war; Strange et al. 2022, Chai et al. 2024). Integrating scenario-based land-use change from paired climate and socioeconomic models into nature recovery systematic conservation planning will be necessary to capture such socioeconomic drivers of land-use changes and ensure nature recovery plans reflect real-world opportunities. The key constraint is the availability of spatial socioeconomic data that reflect appropriate land-use costs (Ban et al. 2013), but open-source land-use models are now widely available that can be parameterized for a given planning region and a range of economic and climate scenarios (Murray-Rust et al. 2014). Nature recovery takes time and therefore considering land value over longer timescales under a range of scenarios is critical for targeting nature recovery in areas that have long-term security.

A major challenge to delivering effective nature recovery is funding the on-the-ground activity, with most landowners requiring at least some level of financial incentive for participation in a nature recovery activity, covering not only payments for operating costs related to nature recovery but also the net income foregone (i.e., opportunity costs associated with loss of agricultural production). This can affect, for example, a landowner's willingness to lease or sell land (Knight et al. 2011) or to engage with nature recovery financing schemes (Reimer and Prokopy 2014). Understanding the funding landscape and budgets for a planning region is crucial at the outset of systematic conservation planning, because they affect the scope of the the conservation goals and the ambition of the targets. Funding for ecosystem restoration activity is increasing globally through a range of initiatives (e.g., Seddon et al. 2021), including payments for ecosystem services programs that offer scalable financial incentives for restoration, facilitated by governments or voluntary markets (United Nations and World Bank 2022). Increasing interest from corporations in developing sustainable supply chains and offsetting the impacts from operations offers a further mechanism to support funding ecosystem restoration activity (United Nations and World Bank 2022).

Information on funding and costs can be incorporated directly into most spatial prioritization algorithms in the conservation assessment stage (box 1, stage 9) but ideally requires quantitative information on funds tied to particular nature recovery activities (e.g., payment per hectare of woodland creation; figure 1). This is easier to implement when funding schemes are well established, defined by a clear set of rules, and where funding is secured and predictable on at least decadal time scales. Unreliable funding effectively expands the restoration gap because areas would be expected to be lost as financial support is removed and alternative land uses are sought to fill the economic gap (figure 2c). Spatial prioritizations must evaluate the consequence of such uncertainty where possible, exploring outcomes across a range of long-term funding scenarios to identify the risk of falling short on targets because of the emergence of funding gaps (e.g., McBride et al. 2007).

### Anticipating responses of conservation features to nature recovery actions

In a nature recovery context, systematic conservation planning is fundamentally about planning for a future state of biodiversity, with solutions depending on the anticipated consequences of nature recovery activity on conservation features. This places considerable importance on understanding and predicting how species or other conservation features are likely to respond to specific nature recovery actions.

Anticipating responses of conservation features to nature recovery actions first requires understanding the types of restoration or creation activity possible at a location (figure 1), with this information necessary to the secondary step of predicting the responses of conservation features to these changes (box 1, stage 5). Potential target and locations for nature recovery actions can be identified most simply using information on historical distributions of natural vegetation under the assumption that appropriate management will restore vegetation to its natural state, and this will provide habitat for historically occurring species (e.g., Westphal et al. 2007, Thomson et al. 2009, Strassburg et al. 2019, Smith et al. 2022). This approach provides a straightforward means of mapping habitat potential across the landscape assuming historical data are available, although it does not consider potential alternative scenarios that might now be favored because of environmental changes that cannot easily be managed (e.g., climate, hydrological flows). Alternatively, approaches that predict the potential to create particular habitats or vegetation types on the basis of current or projected future edaphic and climate conditions (figure 1), including accounting for land use demands under a range of socioeconomic–climate scenarios (Murray-Rust et al. 2014), might be more valuable in landscapes dominated by converted land (e.g., agricultural landscapes of Western Europe) and in still changing environments (e.g., high exposure to climate change). Such information would allow planners to consider scenarios for vegetation or habitat changes that are optimal for meeting the nature recovery targets, deviating from historical baselines where appropriate, and more viable long term (Cordell et al. 2017, Sylvester et al. 2020).

Data on the likely distributional changes of conservation features in response to newly restored or created habitats are a fundamental component of nature recovery systematic conservation planning, being required to determine future priority areas in the conservation assessment stage (box 1, stage 6; figure 1). A range of approaches have been used to date to develop this information for the conservation assessment stage of systematic conservation planning in a nature recovery context, as is illustrated in the examples presented in table 2. Mapping species’ distributions as a function of habitat condition after land-use change requires establishing these functional relationships. The simplest assumption is that species will occupy particular patches of natural vegetation if it is available, accessible, and of sufficient size (Crouzeilles et al. 2015). This may be convenient where current species distributions and species–habitat associations are well known and likely to stay constant under future climate change. Alternatively, predictive models, including correlative species distribution models, can be used to anticipate species’ responses to land-use change (table 2; Thomson et al. 2009, Kujala et al. 2015, Strassburg et al. 2019). A concern is where there is a mismatch between the spatial grain of information required and the spatial grain at which species occurrence data have been sampled. At high spatial resolutions, patterns of species occupancy are driven increasingly by metapopulation dynamics and stochastic processes that are not captured by static correlative species distribution models. Models considering demographic and spatial and temporal processes (Bonnot et al. 2017) and species interactions (McAlpine et al. 2016) are likely to be better than static species distribution models at prediction under novel conditions (Higgins et al. 2020). This may affect prioritization patterns, particularly where restoration gaps are not correctly identified due, for example, to unaccounted for demographic or dispersal constraints.

Following the hierarchy of predictability and the challenges of developing species-specific predictions, some studies have avoided a species-focused approach entirely (table 2), considering species distribution models too uncertain to provide reliable information on the effects of nature recovery actions (Smith et al. 2022). For example, Gilby and colleagues (2021) modeled the relationship between species abundance and the extent of several key environmental features to forecast the effects of restoration. Others have entirely avoided empirical approaches for anticipating responses to nature recovery actions, instead developing scenarios from expert opinion, often linked to vegetation types as the conservation feature (Smith et al. 2022) and assuming different biodiversity benefits of transitioning from one vegetation state to another (Shoo et al. 2021). The choice of approach will depend on the sensitivity of spatial prioritizations to uncertainties in species or vegetation responses, as well as the ecological context (e.g., habitat specificity of focal species, the degree of habitat fragmentation), availability of information to parameterize models (e.g., species occurrence data), along with computational complexity, expertise, and resources.

Planning for nature recovery occurs against the backdrop of ongoing climate change, which is beginning to exert a significant influence on biodiversity, affecting both the value of existing protected area sites and the potential value of new sites for nature recovery (Reside et al. 2018). Existing conservation areas that are known to be effectively managed (e.g., defined through the IUCN Management Effectiveness Tracking Tool; Stolton et al. 2021) and contain high quality environments, would typically provide the core areas from which nature recovery can be built. These areas would be locked into the spatial prioritization at the conservation assessment stage, whereas conservation areas that are not effectively managed or in good condition might be considered among the opportunities for delivering nature recovery in the landscape (i.e., through habitat restoration). Even where conservation areas are currently high quality, climate change impacts to core sites could alter decisions about nature recovery activity and therefore robust analysis of climate change resilience across existing conservation areas is crucial (Baker et al. 2015). Novel approaches to climate modeling are now able to capture changes in species distributions at scales experienced by individuals, such as near the ground or below the canopy (Maclean and Klinges 2021), providing more accurate assessments of climate change exposure and effects on species’ persistence within conservation areas (Maclean and Early 2023). Considering climate change effects in the responses of species to nature recovery activity may be crucial for maximizing the likelihood that nature recovery solutions are robust under a range of climate change scenarios, particularly given recent insights into the importance of fine-scale habitat management for mitigating the effects of climate change on species (Maclean and Early 2023). Accounting for climate change in systematic conservation planning also enables the assessment of the long-term viability of existing sites because of climate change exposure, which is essential for identifying coverage gaps that may emerge (Reside et al. 2018, Critchlow et al. 2022). This can be used to designate the importance of existing conservation areas in the conservation assessment stage by, for example, down weighting the contribution of the most climate change exposed conservation areas to future conservation feature targets (i.e., requiring the solution to compensate for the potential loss of these sites in future with additional sites).

### Conservation assessment for nature recovery

Selecting nature recovery areas requires setting up and solving prioritization problems not dissimilar to those found in prioritizing nature protection. The prioritization for nature recovery must, however, be based on anticipated responses of conservation features to nature recovery activity. This includes predicting the amount of each conservation feature in each planning unit for future time points under a range of nature recovery scenarios, reflecting the likely suitability and availability of land for habitat restoration or creation, ideally capturing temporal changes in vegetation states and habitat quality that reflect restoration gaps, accounting for accessibility on the basis of species’ dispersal traits, and accommodating socioeconomic costs and benefits of nature recovery action in a particular location (figure 1). This creates a number of challenges not encountered when prioritizing for nature protection (box 1, stage 9).

A basic spatial prioritization problem for nature recovery might involve restoring degraded natural vegetation to a known historical state. Because the prioritization problem is simply a binary decision, to restore or not, the problem is somewhat analogous to that encountered in a nature protection context—to protect or not—and this type of problem has been addressed using existing spatial prioritization algorithms with simple modifications to the conservation feature input data (e.g., Westphal et al. 2007, Lethbridge et al. 2010, Carwardine et al. 2015). For example, Thomson and colleagues (2009) used Zonation to target revegetation, including temporal scheduling, by incorporating predicted distribution layers for conservation features at multiple future time points for a given revegetation scenario (table 2). Strassburg and colleagues (2019) extended this problem by exploring how much forest to restore per planning unit in order to maximize ecosystem service benefits while also considering costs, assuming diminishing returns for biodiversity following a species–area relationship. This approach was based on the potential distribution of species as the conservation features, assuming restoration had occurred. McBride and colleagues (2010) showed how the basic Marxan optimization problem could be extended mathematically to include multiple restoration facets, including transitions from different degradation states, spatial dependencies between adjacent sites and incorporating stochastic events (e.g., fire) that might alter outcomes. These extensions do not seem to have been widely adopted for prioritizing restoration, which suggest that although they are mathematically tractable, there are barriers to adoption.

Nature recovery spatial prioritization problems are often more complex than these basic scenarios. For instance, in highly modified landscapes where there is uncertainty about historical environmental states (e.g., much of Western Europe), multiple actions may be possible to implement on a given land parcel dependent on current edaphic, hydrological, and climatic conditions (figure 1). For example, it may be possible to convert the same arable field to species rich grassland, woodland, or wetland, with each potential action benefiting a very different set of species or ecosystem processes and contributing differently toward targets. Marxan with Zones (Watts et al. 2009) extends the Marxan functionality to consider the problem of apportioning the landscape into different conservation zones, therefore optimizing for both the type and spatial distribution of management. Although typically used to assign planning units to particular management strategies given the current distribution of conservation features (e.g., strict, partial, or no protection), the approach can be used to prioritize management actions (or zones). This can be achieved by assigning weights to each conservation feature that represent the contribution of each planning unit to the feature target conditional on the type of habitat created (e.g., which zone the planning unit is assigned to). This type of approach is marginally more complex to setup than a binary restoration problem, requiring additional inputs for each potential nature recovery action (e.g., conservation feature responses to and costs of each action). However, the approach has also not been widely implemented in a nature recovery context (e.g., Smith et al. 2022). Multiaction algorithms have been developed for prioritizing conservation actions aimed at increasing the probability of species persistence, enabling decisions to be made about which suite of actions should be targeted and where to reduce pressures on species across their current range accounting for costs (table 2; Cattarino et al. 2015, Salgado-Rojas et al. 2023).

**Figure 3.**
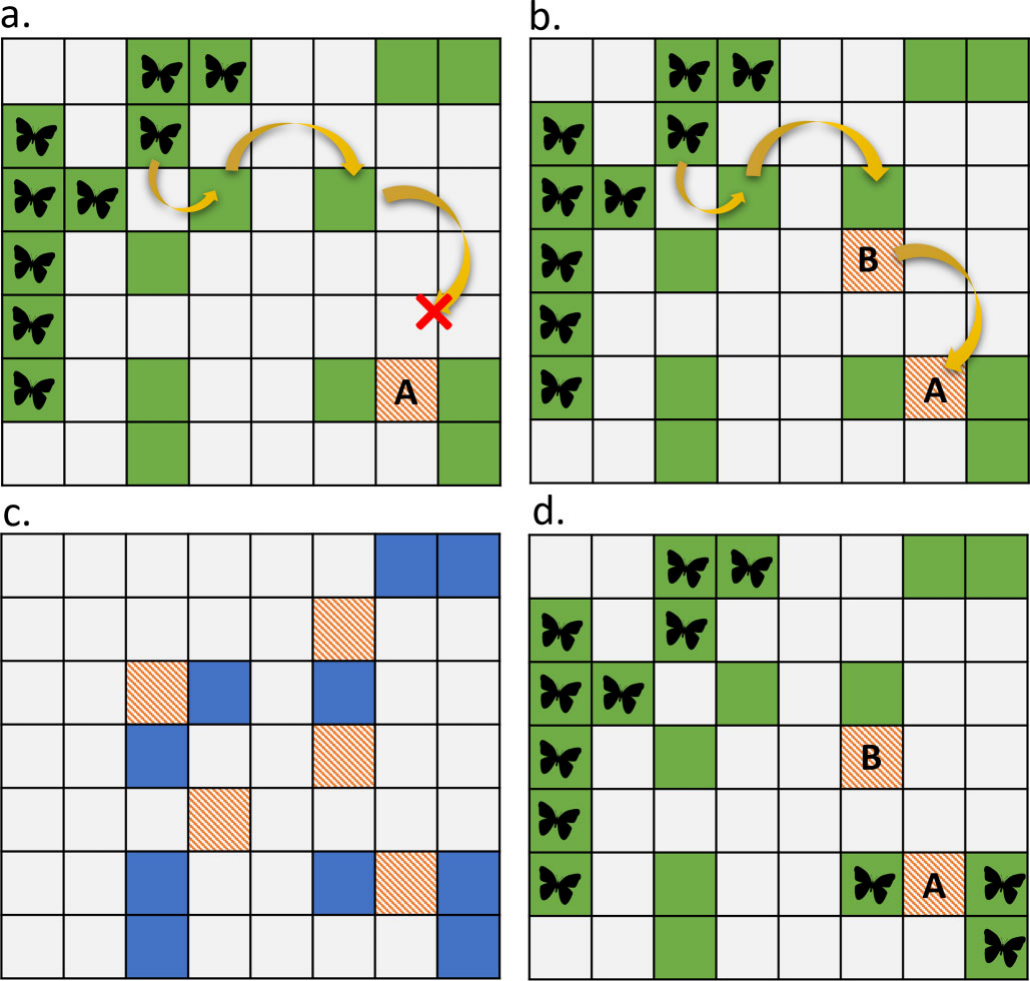
Two examples of important conditional spatial effects to consider in a nature recovery spatial prioritization problem. In panels (a) and (b), the contribution of nature recovery actions at planning unit A to a species’ conservation target is conditional on nature recovery actions occurring in unit B. In panel (a) this is due to dispersal constraints that are resolved in panel (b) through nature recovery actions creating appropriate habitat through which the species can disperse to site A, and regardless of whether B contributes directly to the species’ target. In panels (c) and (d), the contribution of actions at a single planning unit may facilitate occupancy at neighboring sites (e.g., via patch size, connectivity effects) increasing the value of nature recovery actions at a focal site. In panel (c), the hatched planning units are suitable targets for nature recovery actions to benefit a target species, whereas solid planning units have suitable habitat but are currently unoccupied because of patch size or connectivity constraints. In panel (d), nature recovery actions at units A and B permit occupancy of not only A but the surrounding planning units. In a, b, and d, the solid planning units indicate suitable habitat patches, which are occupied or not depending on patch size and connectivity constraints. The unfilled planning units are unsuitable for the species without appropriate nature recovery action, whereas the hatched planning units indicate nature recovery actions to create suitable habitat.

Although these approaches provide a means of spatially prioritizing simple nature recovery scenarios using existing spatial prioritization algorithms, made easier by modern software packages (e.g., Hanson et al. 2024), the use of methods devised for prioritizing nature protection potentially omit important facets of the nature recovery process that could have major consequences for prioritization. For example, although most existing examples prioritize conservation features on the basis of anticipated responses to nature recovery actions, these responses are often static and therefore cannot account for the value of a nature recovery action being conditional on actions conducted elsewhere in the planning region. Figure 3a illustrates this concept, showing that the contribution of nature recovery actions at one planning unit to a species’ conservation target is conditional on nature recovery actions occurring in another unit. This is due to dispersal constraints that are resolved in figure 3b through nature recovery actions creating appropriate habitat through which the species can disperse. Furthermore, nature recovery actions in a planning unit may also facilitate occupancy in neighboring units through effects on patch size or connectivity, therefore multiplying the contribution of a single action to feature targets. Figures 3c and 3d illustrate this concept, showing that nature recovery actions at units A and B permit occupancy of not only A but the surrounding planning units through both patch size and connectivity effects. Recognizing these multiplier effects is important for ensuring that nature recovery actions result in a distribution of patch sizes that allow the persistence of species that are highly sensitive to patch area thresholds. The importance of accounting for such processes in nature recovery spatial prioritization needs to be investigated and may require the continued development of spatial prioritization methods to accommodate additional processes.

The temporal scheduling of nature recovery activities is critical for delivering nature recovery objectives and must consider not only the time scales required for habitat to reach a target state, for target species to colonize these sites, and how suitability might change under climate change (Reside et al. 2018, Dupont-Doare and Alagador 2021) but also the temporal availability of funds required to support nature recovery actions and costs of management (McBride et al. 2010). Temporal scheduling of nature recovery actions has been analyzed by adjusting the feature contribution to a planning unit conditional on when those planning units were restored (e.g., table 2; Shoo et al. 2021) and prioritizing multiple temporal slices simultaneously (Thomson et al. 2009). More complex models that can calculate the value of a nature recovery action conditional on other activities occurring elsewhere in the landscape might have enormous value, shedding light on these spatial and temporal dependencies and their effects on the ordering of interventions (e.g., where occupancy of a site is dependent on the maturation of adjacent habitat). Incorporating temporally varying costs is critical to understand how nature recovery targets will be reached given the sustainability of funding for interventions over decadal timescales (Thomson et al. 2009, Shoo et al. 2021). Temporal scheduling should be considered as part of the *post hoc* analysis of spatial prioritizations prior to implementation, but information to inform decisions must be generated during spatial prioritization and likely calls for novel approaches that can better incorporate the spatial and temporal dynamics of nature recovery.

In this stage, it is important to carefully consider the size of a planning unit and the trade-off between the unit’s size and the accuracy and precision of suggested actions. In a nature protection context, evidence suggests that by including costs and connectivity penalties in the conservation assessment stage, spatial prioritization outputs are less sensitive to the planning unit’s size and shape (Nhancale and Smith 2011). These results may translate to simple nature recovery planning problems, such as binary restoration action problems, but it is not clear how sensitive more complex spatial prioritization problems are to the scale and shape of planning units. An important challenge is where environmental conditions (e.g., soil, microclimate, hydrology) vary over short distances affecting the types of habitat creation opportunities available. Larger planning units are computationally more feasible and may often reflect more accurate knowledge about the distributions of conservation features and costs but may create an implementation challenge where prioritized actions only apply to a fraction of the planning unit. Smaller planning units help link actions more directly to the specific conditions of land parcels and are likely to reflect connectivity barriers for poorly dispersing species better but may add uncertainty where information on conservation features, including anticipated responses to nature recovery actions, must be downscaled to fine resolutions. Therefore, the planning unit size will always involve trade-offs between data uncertainty, computational time, and the degree to which landscapes and ecological processes are sufficiently resolved, and the choice of the planning unit’s size and shape must be tailored to each planning problem.

Conservation assessment in nature recovery systematic conservation planning could become complex, incorporating species’ responses to multiple scenarios for habitat restoration and creation within different socioeconomic–climate contexts while also addressing a range of uncertainties (figure 1). Spatial prioritization algorithms have received considerable attention throughout the development of systematic conservation planning and mathematical optimization tools are well developed. Although it is likely that novel mathematical approaches will continue to help reduce the time taken for algorithms to converge on optimal or near optimal solutions, several other strategies could be considered to reduce complexity. Refining input data is critical, including improving information on the nature recovery potential of land parcels and eliminating socially or economically unfeasible options prior to analysis (Delavenne et al. 2012). The development of regionally specific socioeconomic scenarios for nature recovery (box 1, stage 5) could narrow the range of plausible future conditions to consider. To generate potential rules of thumb for targeting nature recovery actions, lessons from similar landscapes should be examined, and, to achieve this, systematic conservation planning processes and outcomes should be clearly documented and made publicly accessible (McIntosh et al. 2017, Álvarez-Romero et al. 2018, Wintle et al. 2019, Metcalfe et al. 2022).

Finally, the challenge of spatial prioritization for nature recovery has many unique elements, and it is important that mathematical approaches to solving these problems address these elements specifically and are not constrained by existing approaches designed to solve a related but often distinct problem.

### Implementation and monitoring nature recovery actions

The initial decisions on nature recovery priorities, along with the temporal scheduling of interventions and monitoring of nature recovery actions, continue the process of nature recovery systematic conservation planning (box 1, stages 10 and 11). The implementation of nature recovery actions resulting from the conservation assessment stage will encounter legislative, social, and economic barriers that are likely to alter the ultimate realization of the delivery on the ground (Knight et al. 2011, Franks and Emery 2013, Foster and Bell-James 2024). It is important to reiterate that the outputs from conservation assessment are not intended to be prescriptive but are used in the final stage of conservation assessment—stakeholder evaluation—to guide decisions toward effective outcomes. However, the barriers to implementation will inevitably still arise. Most barriers to restoration-type activity occur in the socioeconomic domain and typically relate to insufficient funding, differing priorities of stakeholders, and low political priority, where the time horizons are short but the impacts are realized over longer timescales (Cortina-Segarra et al. 2021). For example, in the United Kingdom’s heavily nature depleted farmed landscapes the highly fragmented nature of land tenue and cultural practices that typically emphasize working independently has proved a barrier to nature recovery despite huge investment in habitat creation because resources have not been effectively targeted (Baker et al. 2012, Baker et al. 2025). Improving nature recovery outcomes requires more effective integration of nature recovery planning with participatory approaches to landscape decisions (e.g., the formation of farmer groups to facilitate joined up decision-making) and legislation and funding mechanisms that facilitate collaboration and cooperation across landscapes (Franks and Emery 2013, Foster and Bell-James 2024, Baker et al. 2025). Decisions must also be made about the degree of intervention required to obtain specific outcomes (i.e., along an active–passive continuum; Chazdon et al. 2024).

**Figure 4.**
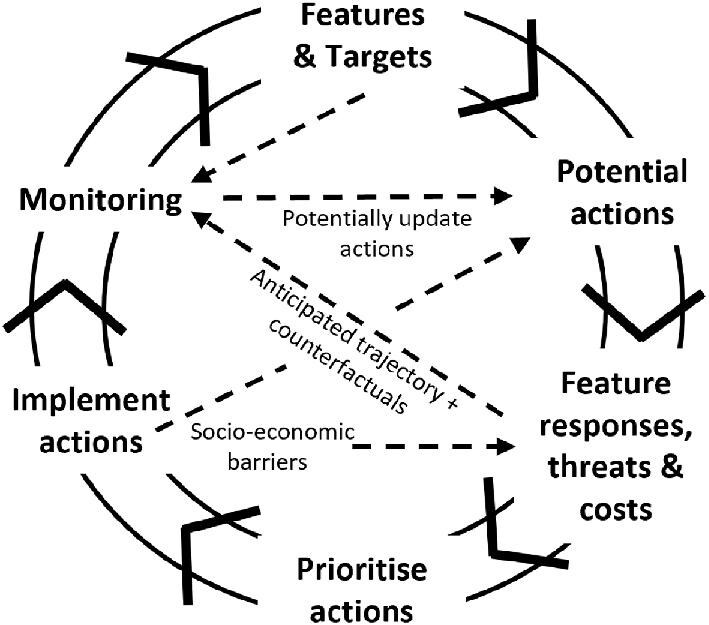
The analytical workflow of systematic conservation planning should be viewed as cyclical and iterative, involving feedback between stages such that monitoring of nature recovery actions implemented on the ground can inform future spatial prioritizations of nature recovery actions and using anticipated responses and counterfactuals to assess progress toward targets.

The long time scales required for nature recovery goals to be realized and the potential variability in the success of nature recovery actions require constant monitoring and periodic reassessment, which may require updating the nature recovery planning through conservation assessment with new input data. The systematic conservation planning steps presented in box 1 could in fact be represented in a circle with feedback occurring between stages to ensure that planning over time reflects outcomes on the ground and is responsive to changes in policy and legislation; this is illustrated in figure 4. Adaptation would also be required to allow for changes in management because of climate change, which requires flexibility in restoration-focused legislation (Foster and Bell-James 2024). Monitoring and active management are vital to ensure targets are achieved, but monitoring must account for time lags in the responses of conservation features to nature recovery actions. Predictive modeling could offer insights into trajectories toward target states enabling *in situ* monitoring to be benchmarked against explicit expectations to track progress and accommodate unsuccessful outcomes (figure 4; Prach et al. 2019). Modeling also provides counterfactuals for assessing nature recovery success (Grace et al. 2021), which is important when there are time lags in meeting targets and ongoing biodiversity declines because of extinction debts and continued species responses to environmental change (figure 2a). Innovations in biodiversity monitoring (e.g., remote audiovisual recorders, satellite remote sensing) are necessary to track progress across vast areas under active nature recovery management with limited monitoring resources. During these environmental transitions, even with sufficient protection of existing populations, extinction debts may reduce source populations of target species, affecting the value of newly created habitat. Understanding the expected temporal dynamics of responses to nature recovery action will be crucial for identifying additional supporting actions that might be required, such as managing existing populations and identifying where assisted colonization of sites might be required in future. Feedback on outcomes is also important for motivating landowners to engage in high-quality environmental management and facilitate a sense of shared responsibility for achieving environmental objectives (Emery and Franks 2012), which is critical, given a deficit in social knowledge about nature recovery is consistently recognized as a major barrier to implementation (Mañas-Navarro et al. 2023). Finally, ensuring that biodiversity metrics used to monitor progress are linked to nature recovery targets from the outset is critical and should be considered in the design of targets as part of the systematic conservation planning process (figure 4).

## Conclusions

Landscape-scale conservation planning principles and decades of systematic conservation planning experience must underpin ecosystem restoration activities if we are to prevent, halt, and reverse the degradation of ecosystems and provide the space for nature to recover and thrive. We propose that systematic conservation planning should be incorporated into policy and guidelines as best practice for targeting resources toward nature recovery. We identify several key challenges to making this an effective strategy for improving the targeting of nature recovery resources:

First, the knowledge and technical skills necessary to use systematic conservation planning must be widely disseminated, ensuring the availability of resources and training to empower local actors in adopting these practices (e.g., Botts et al. 2019). This might involve developing accessible training programs, creating resource hubs, and establishing partnerships to facilitate the transfer of expertise and support to communities engaged in nature recovery initiatives.

Second, Major global data gaps must be addressed, including data on conservation features, habitat restoration and creation opportunities, economic costs, and socioeconomic–climate scenarios. This may be achieved through strategic targeting of *in situ* data collection or through exploiting modeling approaches using existing data to infer, for example, species occurrence across landscapes. Globally, data gaps are likely to be biased toward areas where ecosystem restoration resources are currently needed most and, therefore, resources will require shifting to fill these priority gaps effectively.

Third, Spatial prioritization algorithms are required that are tailored to the unique challenges of prioritizing management actions for nature recovery, where often multiple potential management choices are available within a planning unit and where species responses to nature recovery actions are conditional on actions occurring elsewhere in the landscape that, for example, affect connectivity and patch size. These models should be temporally explicit to account for varied response times of conservation feature to nature recovery actions to guide the temporal scheduling of nature recovery actions and to provide benchmarks and counterfactuals for monitoring progress toward targets.

Fourth, Computational resources needed for systematic conservation planning can be substantial but must become widely available to enable the application of methods in any region of the world. This may involve reducing the computational demands of systematic conservation planning, leveraging innovative computational tools or employing approximate solutions (e.g., via model emulation) derived from the analysis of portfolios of systematic conservation planning solutions and providing access to cloud computing facilities (e.g., Marxan is available in the cloud).

Finally, the monitoring of nature recovery actions must be scaled to provide the data necessary to understand progress toward targets across a representative range of nature recovery projects and locations. This will likely be achieved by taking advantage of developments in remote sensing (e.g., changes in vegetation cover or condition) combined with targeted *in situ* audio and camera trap technology to obtain feature specific information (e.g., changes in taxonomic or functional characteristics).

Achieving ambitious global nature recovery targets requires restoring the fundamental processes supporting biodiversity. Although complete restoration of historical conditions will be unattainable because of modern land use constraints and climate change, strategic targeting of nature recovery actions within spatial and cost limitations are essential. The principles of spatial ecology and decades of systematic conservation planning experience in nature protection highlight the value of strategically prioritizing conservation resource allocation, and now these lessons must be routinely adopted to deliver effective nature recovery.

## Author Biography

David J. Baker (d.baker2@exeter.ac.uk), Kevin J. Gaston, and Ilya M. D. Maclean are affiliated with the Environment and Sustainability Institute, and Kristian Metcalfe is affiliated with the Centre for Ecology and Conservation, at the University of Exeter, in Penryn, Cornwall, England, in the United Kingdom.

## References

[bib1] Aguirre-Gutiérrez J, Stevens N, Berenguer E. 2023. Valuing the functionality of tropical ecosystems beyond carbon. Trends in Ecology and Evolution 38: 1109–1111. 37798181 10.1016/j.tree.2023.08.012

[bib2] Akçakaya HR, Bennett EL, Brooks TM, Grace MK, Heath A, Hedges S, Hilton-Taylor C, Hoffmann M, Keith DA, Long B. 2018. Quantifying species recovery and conservation success to develop an IUCN Green List of species. Conservation Biology 32: 1128–1138. 29578251 10.1111/cobi.13112

[bib3] Álvarez-Romero JG et al. 2018. Research advances and gaps in marine planning: Towards a global database in systematic conservation planning. Biological Conservation 227: 369–382.

[bib4] Anderson P. 1995. Ecological restoration and creation: A review. Biological Journal of the Linnean Society 56: 187–211.

[bib5] Baker DJ, Freeman SN, Grice PV, Siriwardena GM. 2012. Landscape-scale responses of birds to agri-environment management: A test of the English Environmental Stewardship scheme. Journal of Applied Ecology 49: 871–882.

[bib6] Baker DJ, Hartley A, Burgess N, Butchart S, Carr J, Belle E, Willis S. 2015. Assessing climate change impacts for vertebrate fauna across the West African protected area network using regionally appropriate climate projections. Diversity and Distributions 21: 991–1003.

[bib7] Baker DJ, Garnett ST, O'Connor J, Ehmke G, Clarke RH, Woinarski JC, McGeoch MA. 2019. Conserving the abundance of nonthreatened species. Conservation Biology 33: 319–328. 30047186 10.1111/cobi.13197

[bib8] Baker DJ, Maclean IM, Goodall M, Gaston KJ. 2021. Species distribution modelling is needed to support ecological impact assessments. Journal of Applied Ecology 58: 21–26.

[bib9] Baker DJ, Nye C, Wheeler R, Masquelier C, Binner A, Gaston KJ, Heard MS, Lobley M, Smith D, Maclean IMD. 2025. Aligning strategic and participatory approaches to agri-environment scheme design and implementation to enhance nature recovery outcomes. People and Nature 7: 329–345.

[bib10] Ball IR, Possingham HP, Watts M. 2009. Marxan and relatives: Software for spatial conservation prioritisation. Spatial Conservation Prioritisation: Quantitative Methods and Computational Tools 14: 185–196.

[bib11] Ban NC et al. 2013. A social–ecological approach to conservation planning: Embedding social considerations. Frontiers in Ecology and the Environment 11: 194–202.

[bib12] Beyer HL, Dujardin Y, Watts ME, Possingham HP. 2016. Solving conservation planning problems with integer linear programming. Ecological Modelling 328: 14–22.

[bib13] Bond WJ, Stevens N, Midgley GF, Lehmann CER. 2019. The trouble with trees: Afforestation plans for Africa. Trends in Ecology and Evolution 34: 963–965. 31515117 10.1016/j.tree.2019.08.003

[bib14] Bonnot TW, Thompson FR III, Millspaugh JJ. 2017. Dynamic-landscape metapopulation models predict complex response of wildlife populations to climate and landscape change. Ecosphere 8: e01890.

[bib15] Botts EA et al. 2019. Practical actions for applied systematic conservation planning. Conservation Biology 33: 1235–1246. 30912598 10.1111/cobi.13321

[bib16] Brown C, Seo B, Alexander P, Burton V, Chacón-Montalván E, Dunford R, Merkle M, Harrison P, Prestele R, Robinson EL. 2022. Agent-based modeling of alternative futures in the British land use system. Earth's Future 10: e2022EF002905.

[bib17] Carlucci MB, Brancalion PH, Rodrigues RR, Loyola R, Cianciaruso MV. 2020. Functional traits and ecosystem services in ecological restoration. Restoration Ecology 28: 1372–1383.

[bib18] Carwardine J, Hawkins C, Polglase P, Possingham HP, Reeson A, Renwick AR, Watts M, Martin TG. 2015. Spatial priorities for restoring biodiverse carbon forests. BioScience 65: 372–382.

[bib19] Cattarino L, Hermoso V, Carwardine J, Kennard MJ, Linke S. 2015. Multi-action planning for threat management: A novel approach for the spatial prioritization of conservation actions. PLOS ONE 10: e0128027. 26020794 10.1371/journal.pone.0128027 PMC4447389

[bib20] Cava MG, Pilon NA, Ribeiro MC, Durigan G. 2018. Abandoned pastures cannot spontaneously recover the attributes of old-growth savannas. Journal of Applied Ecology 55: 1164–1172.

[bib21] [CBD] Convention on Biological Diversity . 2022. The Kunming–Montreal Global Biodiversity Framework. CBD. www.cbd.int/article/cop15-final-text-kunming-montreal-gbf-221222.

[bib22] Chai L, Liu A, Li X, Guo Z, He W, Huang J, Bai T, Liu J. 2024. Telecoupled impacts of the Russia–Ukraine war on global cropland expansion and biodiversity. Nature Sustainability 7: 432–441.

[bib23] Chan KMA, Shaw MR, Cameron DR, Underwood EC, Daily GC. 2006. Conservation planning for ecosystem services. PLOS Biology 4: e379. 17076586 10.1371/journal.pbio.0040379 PMC1629036

[bib24] Chazdon RL, Falk DA, Banin LF, Wagner M, Wilson SJ, Grabowski RC, Suding KN. 2024. The intervention continuum in restoration ecology: Rethinking the active–passive dichotomy. Restoration Ecology 32: e13535.

[bib25] Cordell S, Questad EJ, Asner GP, Kinney KM, Thaxton JM, Uowolo A, Brooks S, Chynoweth MW. 2017. Remote sensing for restoration planning: How the big picture can inform stakeholders. Restoration Ecology 25: S147–S154.

[bib25a] Cortina-Segarra J et al. 2021. Barriers to ecological restoration in Europe: expert perspectives. Restoration Ecology 29: e13346.

[bib26] Critchlow R, Cunningham CA, Crick HQ, Macgregor NA, Morecroft MD, Pearce-Higgins JW, Oliver TH, Carroll MJ, Beale CM. 2022. Multi-taxa spatial conservation planning reveals similar priorities between taxa and improved protected area representation with climate change. Biodiversity and Conservation 31: 683–702.

[bib27] Crouzeilles R, Beyer HL, Mills M, Grelle CE, Possingham HP. 2015. Incorporating habitat availability into systematic planning for restoration: A species-specific approach for Atlantic Forest mammals. Diversity and Distributions 21: 1027–1037.

[bib28] Daigle RM, Metaxas A, Balbar AC, McGowan J, Treml EA, Kuempel CD, Possingham HP, Beger M. 2020. Operationalizing ecological connectivity in spatial conservation planning with Marxan Connect. Methods in Ecology and Evolution 11: 570–579. 10.1016/j.tree.2022.09.002 36182406

[bib29] Dave R, Saint-Laurent C, Murray L, Antunes Daldegan G, Brouwer R, de Mattos Scaramuzza CA, Raes L, Simonit S, Catapan M, García Contreras G. 2018. Second Bonn Challenge Progress Report: Application of the Barometer in 2019. International Union for Conservation of Nature.

[bib30] Delavenne J, Metcalfe K, Smith RJ, Vaz S, Martin CS, Dupuis L, Coppin F, Carpentier A. 2012. Systematic conservation planning in the eastern English Channel: Comparing the Marxan and Zonation decision-support tools. ICES Journal of Marine Science 69: 75–83.

[bib31] Desmet P, Cowling R. 2004. Using the species–area relationship to set baseline targets for conservation. Ecology and Society 9: 11.

[bib32] Dunn-Capper R, Quintero-Uribe LC, Pereira HM, Sandom CJ. 2023. Diverse approaches to nature recovery are needed to meet the varied needs of people and nature. Sustainability Science (2023): s11625-023-01337-w. 10.1007/s11625-023-01337-w PMC10209942 37363315

[bib33] Dupont-Doare C, Alagador D. 2021. Overlooked effects of temporal resolution choice on climate-proof spatial conservation plans for biodiversity. Biological Conservation 263: 109330.

[bib34] [EC] European Commission . 2020. EU Biodiversity Strategy for 2030: Bringing Nature Back into Our Lives. EC.

[bib34a] Emery SB, Franks JR. 2012. The potential for collaborative agri-environment schemes in England: Can a well-designed collaborative approach address farmers' concerns with current schemes? Journal of Rural Studies 28: 218–231.

[bib35] [FAO] Food and Agriculture Organization of the United Nations . 2021. The State of the World's Land and Water Resources for Food and Agriculture: Systems at Breaking Point. FAO.

[bib36] Fernandes L, Day J, Lewis A, Slegers S, Kerrigan B, Breen D, Cameron D, Jago B, Hall J, Lowe D. 2005. Establishing representative no-take areas in the Great Barrier Reef: Large-scale implementation of theory on marine protected areas. Conservation Biology 19: 1733–1744.

[bib36a] Foster R, Bell-James J. 2024. Legal barriers and enablers to upscaling ecological restoration. Restoration Ecology 32: e14203.

[bib37] Franks JR, Emery SB. 2013. Incentivising collaborative conservation: Lessons from existing environmental Stewardship Scheme options. Land Use Policy 30: 847–862.

[bib38] Gann GD et al. 2019. International principles and standards for the practice of ecological restoration. Restoration Ecology 27: S1–S46.

[bib39] Garibaldi LA et al. 2021. Working landscapes need at least 20% native habitat. Conservation Letters 14: e12773.

[bib40] Gilby BL, Olds AD, Brown CJ, Connolly RM, Henderson CJ, Maxwell PS, Schlacher TA. 2021. Applying systematic conservation planning to improve the allocation of restoration actions at multiple spatial scales. Restoration Ecology 29: e13403.

[bib41] Gordon A, Simondson D, White M, Moilanen A, Bekessy SA. 2009. Integrating conservation planning and land use planning in urban landscapes. Landscape and Urban Planning 91: 183–194.

[bib42] Grace M, Akçakaya HR, Bennett E, Hilton-Taylor C, Long B, Milner-Gulland EJ, Young R, Hoffmann M. 2019. Using historical and palaeoecological data to inform ambitious species recovery targets. Philosophical Transactions of the Royal Society B 374: 20190297. 10.1098/rstb.2019.0297 PMC6863500 31679497

[bib43] Grace MK, Akçakaya HR, Bull JW, Carrero C, Davies K, Hedges S, Hoffmann M, Long B, Lughadha EMN, Martin GM. 2021. Building robust, practicable counterfactuals and scenarios to evaluate the impact of species conservation interventions using inferential approaches. Biological Conservation 261: 109259.

[bib44] Hanson JO, Rhodes JR, Riginos C, Fuller RA. 2017. Environmental and geographic variables are effective surrogates for genetic variation in conservation planning. Proceedings of the National Academy of Sciences 114: 12755–12760. 10.1073/pnas.1711009114 PMC5715761 29087942

[bib45] Hanson JO, Schuster R, Strimas-Mackey M, Morrell N, Edwards BPM, Arcese P, Bennett JR, Possingham HP. 2024. Systematic conservation prioritization with the prioritizr R package. Conservation Biology 39: e14376. 39268847 10.1111/cobi.14376 PMC11780203

[bib46] Harris JA, Hobbs RJ, Higgs E, Aronson J. 2006. Ecological restoration and global climate change. Restoration Ecology 14: 170–176.

[bib47] Higgins SI, Larcombe MJ, Beeton NJ, Conradi T, Nottebrock H. 2020. Predictive ability of a process-based versus a correlative species distribution model. Ecology and Evolution 10: 11043–11054. 33144947 10.1002/ece3.6712 PMC7593166

[bib48] Hua F, Bruijnzeel LA, Meli P, Martin PA, Zhang J, Nakagawa S, Miao X, Wang W, McEvoy C, Peña-Arancibia JL. 2022. The biodiversity and ecosystem service contributions and trade-offs of forest restoration approaches. Science 376: 839–844. 35298279 10.1126/science.abl4649

[bib49] [IPBES] Intergovernmental Science-Policy Platform on Biodiversity and Ecosystem Services . 2019. Summary for Policy Makers of the Global Assessment Report on Biodiversity and Ecosystem Services of the Intergovernmental Science-Policy Platform on Biodiversity and Ecosystem Services. IPBES.

[bib50] Iversen SV, Convery I, Mansfield L, Holt CD. 2022. Why understanding stakeholder perspectives and emotions is important in upland woodland creation: A case study from Cumbria, UK. Land Use Policy 114: 105929.

[bib51] Jaureguiberry P et al. 2022. The direct drivers of recent global anthropogenic biodiversity loss. Science Advances 8: eabm9982. 36351024 10.1126/sciadv.abm9982 PMC9645725

[bib52] Kapás RE, Kimberley A, Cousins SA. 2023. Grassland species colonization of a restored grassland on a former forest varies in short-term success but is facilitated by greater functional connectivity. Nordic Journal of Botany 2024: e03762.

[bib53] Klein CJ, Steinback C, Watts M, Scholz AJ, Possingham HP. 2010. Spatial marine zoning for fisheries and conservation. Frontiers in Ecology and the Environment 8: 349–353.

[bib54] Knight AT, Driver A, Cowling RM, Maze K, Desmet PG, Lombard AT, Rouget M, Botha MA, Boshoff AF, Castley JG. 2006. Designing systematic conservation assessments that promote effective implementation: Best practice from South Africa. Conservation Biology 20: 739–750. 16909567 10.1111/j.1523-1739.2006.00452.x

[bib55] Knight AT, Grantham HS, Smith RJ, McGregor GK, Possingham HP, Cowling RM. 2011. Land managers’ willingness-to-sell defines conservation opportunity for protected area expansion. Biological Conservation 144: 2623–2630.

[bib56] Kujala H, Whitehead AL, Morris WK, Wintle BA. 2015. Towards strategic offsetting of biodiversity loss using spatial prioritization concepts and tools: A case study on mining impacts in Australia. Biological Conservation 192: 513–521.

[bib57] Kukkala AS, Moilanen A. 2013. Core concepts of spatial prioritisation in systematic conservation planning. Biological Reviews 88: 443–464. 23279291 10.1111/brv.12008 PMC3654170

[bib58] Laughlin DC, Strahan RT, Moore MM, Fulé PZ, Huffman DW, Covington WW. 2017. The hierarchy of predictability in ecological restoration: Are vegetation structure and functional diversity more predictable than community composition? Journal of Applied Ecology 54: 1058–1069.

[bib59] Laughlin DC, Chalmandrier L, Joshi C, Renton M, Dwyer JM, Funk JL. 2018. Generating species assemblages for restoration and experimentation: A new method that can simultaneously converge on average trait values and maximize functional diversity. Methods in Ecology and Evolution 9: 1764–1771.

[bib60] Leclère D et al. 2020. Bending the curve of terrestrial biodiversity needs an integrated strategy. Nature 585: 551–556. 32908312 10.1038/s41586-020-2705-y

[bib61] Lethbridge MR, Westphal MI, Possingham HP, Harper ML, Souter NJ, Anderson N. 2010. Optimal restoration of altered habitats. Environmental Modelling and Software 25: 737–746.

[bib62] Maclean IM, Early R. 2023. Macroclimate data overestimate range shifts of plants in response to climate change. Nature Climate Change 13: 484–490.

[bib63] Maclean IM, Klinges DH. 2021. Microclimc: A mechanistic model of above, below, and within-canopy microclimate. Ecological Modelling 451: 109567.

[bib63a] Mañas-Navarro JJ, Aledo A, Ortiz G, Cortina-Segarra J. 2023. Unravelling social barriers in ecological restoration decision-making: A network analysis approach. Land Degradation & Development 34: 4897–4911.

[bib64] Margules CR, Pressey RL. 2000. Systematic conservation planning. Nature 405: 243–253. 10821285 10.1038/35012251

[bib65] McAlpine C, Catterall CP, Nally RM, Lindenmayer D, Reid JL, Holl KD, Bennett AF, Runting RK, Wilson K, Hobbs RJ. 2016. Integrating plant-and animal-based perspectives for more effective restoration of biodiversity. Frontiers in Ecology and the Environment 14: 37–45.

[bib66] McBride MF, Wilson KA, Bode M, Possingham HP. 2007. Incorporating the effects of socioeconomic uncertainty into priority setting for conservation investment. Conservation Biology 21: 1463–1474. 18173470 10.1111/j.1523-1739.2007.00832.x

[bib67] McBride MF, Wilson KA, Burger J, Fang Y-C, Lulow M, Olson D, O'Connell M, Possingham HP. 2010. Mathematical problem definition for ecological restoration planning. Ecological Modelling 221: 2243–2250.

[bib68] McIntosh EJ, Pressey RL, Lloyd S, Smith RJ, Grenyer R. 2017. The impact of systematic conservation planning. Annual Review of Environment and Resources 42: 677–697.

[bib69] McIntosh EJ, Chapman S, Kearney SG, Williams B, Althor G, Thorn JP, Pressey RL, McKinnon MC, Grenyer R. 2018. Absence of evidence for the conservation outcomes of systematic conservation planning around the globe: A systematic map. Environmental Evidence 7: 1–23.

[bib70] Metcalfe K, Vaz S, Engelhard GH, Villanueva MC, Smith RJ, Mackinson S. 2015. Evaluating conservation and fisheries management strategies by linking spatial prioritization software and ecosystem and fisheries modelling tools. Journal of Applied Ecology 52: 665–674.

[bib71] Metcalfe K et al. 2022. Fulfilling global marine commitments: Lessons learned from Gabon. Conservation Letters 15: e12872.

[bib72] Mikołajczak KM, Jones N, Sandom CJ, Wynne-Jones S, Beardsall A, Burgelman S, Ellam L, Wheeler HC. 2022. Rewilding: The farmers’ perspective. Perceptions and attitudinal support for rewilding among the English farming community. People and Nature 4: 1435–1449.

[bib73] Mu Y, Guo Y, Li X, Li P, Bai J, Linke S, Cui B. 2022. Cost-effective integrated conservation and restoration priorities by trading off multiple ecosystem services. Journal of Environmental Management 320: 115915. 35952567 10.1016/j.jenvman.2022.115915

[bib74] Murray-Rust D, Robinson DT, Guillem E, Karali E, Rounsevell M. 2014. An open framework for agent based modelling of agricultural land use change. Environmental Modelling and Software 61: 19–38.

[bib75] Naidoo R, Balmford A, Ferraro P, Polasky S, Ricketts T, Rouget M. 2006. Integrating economic costs into conservation planning. Trends in Ecology and Evolution 21: 681–687. 17050033 10.1016/j.tree.2006.10.003

[bib76] Nhancale BA, Smith RJ. 2011. The influence of planning unit characteristics on the efficiency and spatial pattern of systematic conservation planning assessments. Biodiversity and Conservation 20: 1821–1835.

[bib77] Nielsen ES, Hanson JO, Carvalho SB, Beger M, Henriques R, Kershaw F, Von Der Heyden S. 2023. Molecular ecology meets systematic conservation planning. Trends in Ecology and Evolution 38: 143–155. 36210287 10.1016/j.tree.2022.09.006

[bib78] Noss R, Nielsen S, Vance-Borland K. 2009. Prioritizing ecosystems, species, and sites for restoration. Pages 158–171 in Moilanen A, Wilson KA, Possingham HP, eds. Spatial Conservation Prioritization: Quantitative Methods and Computational Tools. Oxford University Press.

[bib79] O'Bryan CJ, Rhodes JR, Osunkoya OO, Lundie-Jenkins G, Mudiyanselage NA, Sydes T, Calvert M, McDonald-Madden E, Bode M. 2023. Setting conservation priorities in multi-actor systems. BioScience 73: 522–532. 39634923 10.1093/biosci/biad046 PMC11616722

[bib80] Possingham HP, Bode M, Klein CJ. 2015. Optimal conservation outcomes require both restoration and protection. PLOS Biology 13: e1002052. 25625277 10.1371/journal.pbio.1002052 PMC4308106

[bib81] Potapov P, Turubanova S, Hansen MC, Tyukavina A, Zalles V, Khan A, Song XP, Pickens A, Shen Q, Cortez J. 2022. Global maps of cropland extent and change show accelerated cropland expansion in the twenty-first century. Nature Food 3: 19–28. 37118483 10.1038/s43016-021-00429-z

[bib82] Prach K, Durigan G, Fennessy S, Overbeck GE, Torezan JM, Murphy SD. 2019. A primer on choosing goals and indicators to evaluate ecological restoration success. Restoration Ecology 27: 917–923.

[bib83] Pressey RL, Bottrill MC. 2009. Approaches to landscape-and seascape-scale conservation planning: Convergence, contrasts and challenges. Oryx 43: 464–475.

[bib84] Pressey RL, Cowling RM, Rouget M. 2003. Formulating conservation targets for biodiversity pattern and process in the Cape Floristic Region, South Africa. Biological Conservation 112: 99–127.

[bib85] Reimer AP, Prokopy LS. 2014. Farmer participation in US Farm Bill conservation programs. Environmental Management 53: 318–332. 24114348 10.1007/s00267-013-0184-8

[bib86] Reside AE, Butt N, Adams VM. (2018). Adapting systematic conservation planning for climate change. Biodiversity and Conservation 27: 1–29.

[bib87] Rodrigues ASL, Brooks TM. 2007. Shortcuts for biodiversity conservation planning: The effectiveness of surrogates. Annual Review of Ecology, Evolution, and Systematics 38: 713–737.

[bib87a] Rondinini C, Chiozza F. 2010. Quantitative methods for defining percentage area targets for habitat types in conservation planning. Biological Conservation 143: 1646–1653.

[bib88] Salgado-Rojas J, Hermoso V, Álvarez-Miranda E. 2023. prioriactions: Multi-action management planning in R. Methods in Ecology and Evolution 16: 14220.

[bib89] Seddon N, Turner B, Berry P, Chausson A, Girardin CA. 2019. Grounding nature-based climate solutions in sound biodiversity science. Nature Climate Change 9: 84–87.

[bib90] Seddon N, Smith A, Smith P, Key I, Chausson A, Girardin C, House J, Srivastava S, Turner B. 2021. Getting the message right on nature-based solutions to climate change. Global Change Biology 8: 1518–1546. 10.1111/gcb.15513 33522071

[bib91] Shoo LP, Catterall CP, Beyer HL, Cockbain P, Duncan M, Robson T, Roche D, Taylor H, White Z, Wilson K. 2021. Smart allocation of restoration funds over space and time. Ecological Applications 31: e02448. 34514663 10.1002/eap.2448

[bib92] Smith RJ, Di Minin E, Linke S, Segan DB, Possingham HP. 2010. An approach for ensuring minimum protected area size in systematic conservation planning. Biological Conservation 143: 2525–2531.

[bib93] Smith RJ, Cartwright SJ, Fairbairn AC, Lewis DC, Gibbon GE, Stewart CL, Sykes RE, Addison PF. 2022. Developing a nature recovery network using systematic conservation planning. Conservation Science and Practice 4: e578.

[bib94] Stolton S, Dudley N, Hockings M. 2021. METT Handbook: A Guide to Using the Management Effectiveness Tracking Tool (METT). World Wildlife Fund.

[bib95] Strange N et al. Policy responses to the Ukraine crisis threaten European biodiversity. Nature Ecology and Evolution 6: 1048–1049. 10.1038/s41559-022-01786-z 35606519

[bib96] Strassburg BB, Beyer HL, Crouzeilles R, Iribarrem A, Barros F, de Siqueira MF, Sanchez-Tapia A, Balmford A, Sansevero JBB, Brancalion PHS. 2019. Strategic approaches to restoring ecosystems can triple conservation gains and halve costs. Nature Ecology and Evolution 4: 765–765. 10.1038/s41559-018-0743-8 30568285

[bib97] Strassburg BB et al. 2020. Global priority areas for ecosystem restoration. Nature 586: 724–729. 33057198 10.1038/s41586-020-2784-9

[bib98] Sylvester J, Valencia J, Verchot LV, Chirinda N, Romero Sanchez MA, Quintero M, Castro-Nunez A. 2020. A rapid approach for informing the prioritization of degraded agricultural lands for ecological recovery: A case study for Colombia. Journal for Nature Conservation 58: 125921.

[bib99] Thomson JR, Moilanen A, Vesk P, Bennett AF, Nally RM. 2009. Where and when to revegetate: A quantitative method for scheduling landscape reconstruction. Ecological Applications 19: 817–828. 19544726 10.1890/08-0915.1

[bib100] United Nations and World Bank. 2022. Scaling up Ecosystem Restoration Finance: A Stocktake Report. Wold Bank. 10.1596/38311.

[bib101] Villarreal-Rosas J, Sonter LJ, Runting RK, López-Cubillos S, Dade MC, Possingham HP, Rhodes JR. 2020. Advancing systematic conservation planning for ecosystem services. Trends in Ecology and Evolution 35: 1129–1139. 32977982 10.1016/j.tree.2020.08.016

[bib102] Watson JE, Grantham HS, Wilson KA, Possingham HP. 2011. Systematic conservation planning: Past, present, and future. Pages 136–160 in Ladle RJ, Whittaker RJ, eds. Conservation Biogeography. Wiley.

[bib103] Watts ME, Ball IR, Stewart RS, Klein CJ, Wilson K, Steinback C, Lourival R, Kircher L, Possingham HP. 2009. Marxan with zones: Software for optimal conservation based land-and sea-use zoning. Environmental Modelling and Software 24: 1513–1521.

[bib104] Westphal MI, Field SA, Possingham HP. 2007. Optimizing landscape configuration: A case study of woodland birds in the Mount Lofty Ranges, South Australia. Landscape and Urban Planning 81: 56–66.

[bib105] Winfree R, Fox JW, Williams NM, Reilly JR, Cariveau DP. 2015. Abundance of common species, not species richness, drives delivery of a real-world ecosystem service. Ecology Letters 18: 626–635. 25959973 10.1111/ele.12424

[bib106] Wintle BA, Kujala H, Whitehead A, Cameron A, Veloz S, Kukkala A, Moilanen A, Gordon A, Lentini PE, Cadenhead NC. 2019. Global synthesis of conservation studies reveals the importance of small habitat patches for biodiversity. Proceedings of the National Academy of Sciences 116: 909–914. 10.1073/pnas.1813051115 PMC6338828 30530660

[bib107] [WWF] World Wildlife Fund . 2022. Living Planet Report 2022: Building a Nature-Positive Society. WWF.

[bib108] Wynne-Jones S, Strouts G, Holmes G. 2018. Abandoning or reimagining a cultural heartland? Understanding and responding to rewilding conflicts in Wales: The case of the Cambrian Wildwood. Environmental Values 27: 377–403.

